# Interleukin-3 protects against viral pneumonia in sepsis by enhancing plasmacytoid dendritic cell recruitment into the lungs and T cell priming

**DOI:** 10.3389/fimmu.2023.1140630

**Published:** 2023-02-22

**Authors:** Alan Bénard, Frederik J. Hansen, Florian Uhle, Bettina Klösch, Franziska Czubayko, Anke Mittelstädt, Anne Jacobsen, Paul David, Malgorzata J. Podolska, Anna Anthuber, Izabela Swierzy, Dominik Schaack, Petra Mühl-Zürbes, Alexander Steinkasserer, Michael Weyand, Markus A. Weigand, Thorsten Brenner, Christian Krautz, Robert Grützmann, Georg F. Weber

**Affiliations:** ^1^ Department of Surgery, Friedrich-Alexander University (FAU) Erlangen-Nürnberg and Universitätsklinikum Erlangen, Erlangen, Germany; ^2^ Department of Anesthesiology, Heidelberg University Hospital, Heidelberg, Germany; ^3^ Department of Immune Modulation, Friedrich-Alexander University (FAU) Erlangen-Nürnberg and Universitätsklinikum Erlangen, Erlangen, Germany; ^4^ Department of Cardiac Surgery, Friedrich-Alexander University (FAU) Erlangen-Nürnberg and Universitätsklinikum Erlangen, Erlangen, Germany; ^5^ Department of Anesthesiology and Intensive Care Medicine, University Hospital Essen, University Duisburg-Essen, Essen, Germany

**Keywords:** interleukin-3, sepsis, viral pneumonia, plasmacytoid dendritic cells, T cell priming

## Abstract

**Rationale:**

Sepsis, a global health burden, is often complicated by viral infections leading to increased long-term morbidity and mortality. Interleukin-3 (IL-3) has been identified as an important mediator amplifying acute inflammation in sepsis; however, its function in the host response to viral infections during sepsis remains elusive.

**Objectives:**

To investigate the role of IL-3 during viral pneumonia in sepsis.

**Methods:**

We included septic patients from two different cohorts and used *in vitro* and *in vivo* assays. The obtained data were substantiated using a second model (SARS-CoV-2 infections).

**Measurements and main results:**

Low plasma IL-3 levels were associated with increased herpes simplex virus (HSV) airway infections in septic patients, resulting in reduced overall survival. Likewise, *Il-3*-deficient septic mice were more susceptible to pulmonary HSV-1 infection and exhibited higher pulmonary inflammation than control mice. Mechanistically, IL-3 increases innate antiviral immunity by promoting the recruitment of circulating plasmacytoid dendritic cells (pDCs) into the airways and by enhancing pDC-mediated T cell activation upon viral stimulation. Interestingly, the ability of IL-3 to improve adaptive immunity was confirmed in patients with SARS-CoV-2 infections.

**Conclusion:**

Our study identifies IL-3 as a predictive disease marker for viral reactivation in sepsis and reveals that IL-3 improves antiviral immunity by enhancing the recruitment and the function of pDCs.

## Introduction

Sepsis is a life-threatening organ dysfunction caused by a dysregulated host response to infection (1) resulting approximatively in 20% of all-cause deaths worldwide (WHO). Successful treatment of patients in the excessive inflammatory phase improves short-term outcome but gives rise to a profound immunosuppressive state (2–4). This immunosuppressive phase is characterized by immune cell apoptosis, elevated levels of anti-inflammatory cytokines or T-cell exhaustion (3, 4) and results in increased sensitivity to nosocomial infections and viral reactivation leading to poorer outcome and increased long-term mortality (5–8). Recent studies reported that patients in intensive care units (ICU) exhibited a cumulative incidence of viremia from 10% to 53% with at least one detectable (plasma or respiratory secretion) virus such as cytomegalovirus, Epstein–Barr virus, human herpesvirus 6 or herpes simplex virus-1 (HSV-1) in 80% of patients admitted to the ICU for septic shock (9–11). Interestingly, HSV-1 was described to be the most frequently isolated pathogen in the lungs of patients with severe respiratory distress (12) and being related to poor outcome in critically ill patients (13). In sepsis, HSV-1 showed a 25-fold cumulative incidence rate during the first week and patient positive for HSV had increased opportunistic bacterial infections and increased ICU length of stays compared to viral negative patients (9, 10). Thus, it is a health priority to identify septic patients at risk to develop viral infections.

Recently, interleukin 3 (IL-3) has been identified as a predictive marker for severity and outcome during SARS-CoV-2 infections, a disease exhibiting systemic cytokine profiles similar to those observed in cytokine release syndromes (14, 15). IL-3 is a hematopoietic growth factor produced mainly by immune cells (16–19) that plays a key role during inflammatory diseases by promoting either the survival, the differentiation, the proliferation or the recruitment of leukocytes (19–22). During viral pneumonia, IL-3 improves innate antiviral immunity by promoting plasmacytoid dendritic cells (pDC) recruitment into the lungs (21). In sepsis, IL-3 has been described as a central upstream mediator involved in the amplification of the acute phase by inducing emergency haematopoiesis (19). However, the impact of IL-3 in the subsequent immunosuppressive phase, in which secondary viral infections and viral re-activations occur, remains unclear.

pDCs are immune cells playing critical roles in viral immunity (23) that are known to produce large amount of type 1 interferons after sensing viral RNA and DNA (24). Moreover, pDCs are an essential link between innate and adaptive viral immunity through their capacity to prime T cells (23, 25) as shown by their ability to control T cell responses to chronic lymphocytic choriomeningitis virus (LCMV) infection (26) as well as to cross-present viral antigen from influenza exposed cells (27). In sepsis, patients with profound and persistent decrease of circulating pDCs exhibited an increased risk to develop ICU-acquired infection (28) as well as increased mortality (29) suggesting that pDCs play an important role during viral pneumonia in sepsis. The α chain of the IL-3 receptor is highly expressed on human pDCs compared to other leukocytes whereas murine pDCs do not express it (23). IL-3 was described to promote pDC survival, to increase the ability of pDCs to prime T cells and to synergize with TLRs to allow mTORC1 activation and the associated metabolic changes necessary for cellular activation (30–32). However, the effect of IL-3 on pDCs during viral infection is still poorly understood.

The aim of our study was to investigate the role of IL-3 during viral infections in sepsis. We included septic patients from two different study cohorts and designed a mouse model of secondary viral pneumonia in sepsis. First, we showed that IL-3 is associated with protection against viral pneumonia during sepsis in both humans and mice. Second, we determined that IL-3 promotes the recruitment of pDCs into the lungs of CLP mice during viral pneumonia and that the *ex vivo* stimulation of human circulating pDCs by IL-3 results in increased T cell activation upon viral infection. Finally, the ability of IL-3 to improve adaptive immunity was confirmed by analysing patients with SARS-CoV-2 infections.

## Results

### Interleukin-3 is associated with reduced pulmonary viral infections in septic patients

**Figure 1:**
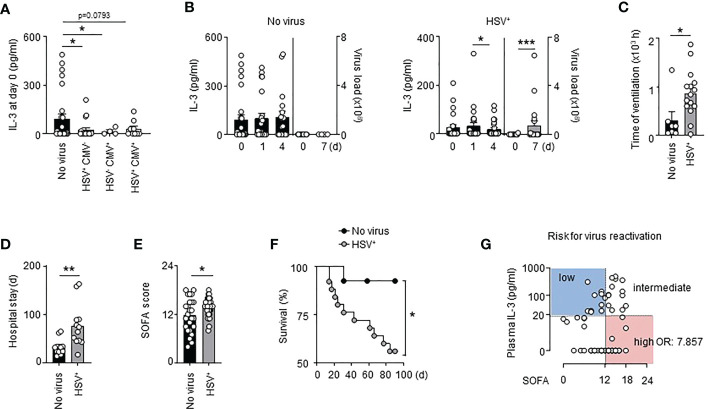
Interleukin-3 is associated with reduced viral infections in septic patients. **(A)** Levels of IL-3 in the serum of septic patients positive for HSV (n=19), CMV (n=4) or both (n=9) in the tracheal secretion or in septic patient without viral pneumonia (n=27). **(B)** Levels of IL-3 in the serum and viral load in the BALF of septic HSV^+^ patients or septic patients without viral pneumonia at days 0, 1 and 4 after the onset of sepsis (n=55). **(C)** Time under ventilation of septic HSV^+^ patients or septic patients without viral pneumonia in the VISS trial (n=55). **(D)** Hospital stay of septic HSV^+^ patients or septic patients without viral pneumonia in the VISS trial (n=55). **(E)** SOFA score of septic HSV^+^ patients or septic patients without viral pneumonia in the VISS trial (n=55). **(F)** Survival curve of septic HSV^+^ patients or septic patients without viral pneumonia between the 14^th^ and 90^th^ day following sepsis onset (n=38). Data were analysed by Log-rank (Mantel-Cox) test. **(G)** Analysis of plasma IL-3 levels and SOFA score defining risk groups to develop secondary viral infections (low and intermediate vs high; OR: 7.857; 95% CI: 2.607 – 23.682) in the pooled VISS and SEPICER sepsis cohorts (n=72). Data are mean ± s.e.m., *P < 0.05, **P < 0.01, ***P < 0.001, Wilcoxon test or unpaired, 2-tailed Student’s t test using Welch’s correction for unequal variances was used.

We retrospectively analysed a cohort of septic patients (VISS trial (33)) in which the presence of HSV-1 and CMV in the tracheal secretion was assessed (**Table S1**). We found that septic patients either positive for HSV-1, CMV or both in the bronchoalveolar lavage fluid (BALF) showed lower plasma IL-3 levels at the onset of sepsis than septic patients in which no virus was detected in the BALF during the first 28 days of sepsis (**Figure 1A**). Compared to controls, septic patients positive for HSV-1 (HSV^+^) displayed reduced IL-3 plasma levels over a period of 4 days after the onset of sepsis, with lowest levels at day 4 (**Figure 1B**). This reduction was associated with increased viral copy numbers in the BALF 7 days after sepsis onset (**Figure 1B**). Septic HSV^+^ patients exhibited an increased duration of mechanical ventilation (**Figure 1C**) an increased length of hospital stay (**Figure 1D**), and showed higher SOFA scores (**Figure 1E**) resulting in increased mortality beyond day 14 after sepsis onset (**Figure 1F**), a time point in which septic patients exhibited markers of immunosuppression compared to healthy donors such as increased plasma IL-10 levels, unaltered plasma TNFα levels and reduced monocytic HLA-DR (**Figure S1**). The association between IL-3 and pulmonary viral infections in septic patients was confirmed using a prospective cohort (SEPICER trial; **Table S2**). Indeed, septic patients with either primary (SARS-CoV-2) lung viral infections or viral reactivation (HSV-1) showed similarly decreased plasma IL-3 levels (**Figure S2**) when compared to septic patients without viral pneumonia. Thus, our data confirm the knowledge that pulmonary viral infections during sepsis is associated with increased mortality and suggest that IL-3 may protect within this context.

### Interleukin-3 is a predictive marker for viral reactivation during sepsis

It was previously described that 88% of septic shock patients had at least one viremia event during the first week (9). As well, 76% (25/33) of septic patients that developed lung viral infections in the VISS cohort exhibited viral reactivation in BALF during the first week of sepsis indicating that virus reactivation occurs during the early stage of sepsis. To identify patients at risk for virus reactivation, we decided to pool the two cohorts (VISS and SEPICER). We included only septic patients from the two cohorts for which blood samples were collected during the first week of sepsis. We also excluded septic patients with primary lung viral infections. Using a minimal *p*-value approach, we observed that patients with SOFA scores ≥12 had the highest risk for virus reactivation in lungs (**Table S3**), this association remaining significant after adjusting for prognostic parameters in multivariate analysis (**Table S3**). Plasma IL-3 levels ≥20 pg/ml at admission was associated with a better outcome during SARS-CoV-2 infections (21). Likewise, septic patients with plasma IL-3 levels < 20 pg/ml showed increased risk for virus reactivation in lungs (**Table S4**). Thus, the correlation of plasma IL-3 levels and SOFA score allowed to identify different groups at risk: patients with IL-3 levels ≥20 pg/ml and SOFA score <12 or ≥12 had a low to intermediate risk for virus reactivation in lungs whereas patients with IL-3 levels <20 pg/ml and SOFA score < 12 or ≥ 12 had an intermediate to high risk for virus reactivation in lungs during sepsis (OR: 7.857; 95% CI: 2.607 – 23.682) (**Figures 1G**, **S3** and **Table S5**). Collectively, our results suggest that the combination of plasma IL-3 levels and SOFA score may be an early predictive marker to identify patients at risk for virus reactivation during sepsis.

### Interleukin-3-deficient mice are more susceptible to viral pneumonia during sepsis

**Figure 2:**
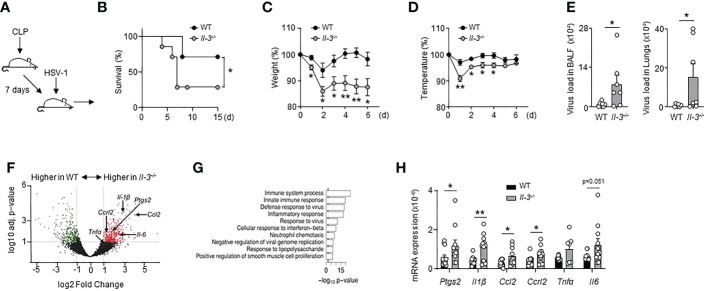
Interleukin-3 protects against pulmonary viral infection during sepsis. **(A–H)** Sub-lethal sepsis was induced in WT and *Il-3^-/-^* mice using the CLP model, and 7 days later, mice were intranasally infected with 1.5x10^7^ PFU of HSV-1 during 3 days. **(A)** Schematic representation of sepsis induction and infection. **(B)** Survival curve (n=7/group). Data were analysed by Log-rank (Mantel-Cox) test. **(C)** Percentage of weight loss (n=8). **(D)** Body temperature percentage (n=8). **(E)** Viral load in the BALF and lungs (n=8). **(F)** Volcano plot representation showing genes increased in the lungs of CLP WT mice compared with CLP *Il-3^-/-^* mice (n=5). **(G)** Top 10 over-represented GO term results ordered according to their p-value for the full set of differentially expressed genes identified by DESeq2 (*Il-3^-/-^* vs. WT). **(H)** Relative mRNA expression of genes identified by volcano plot in the lungs of septic WT or *Il-3^-/-^* mice (n=13). Data are mean ± s.e.m., *P < 0.05, **P < 0.01, unpaired, 2-tailed Student’s t test using Welch’s correction for unequal variances was used.

To determine if IL-3 protects from viral infections during the immunosuppressive phase of sepsis, we designed a mouse model of poly-microbial sepsis (34) in which wild-type (WT) and *Il-3^-/-^* mice were subjected to sub-lethal caecal ligation and puncture (CLP), followed by intranasal (i.n.) HSV-1 administration 7 days after CLP (**Figure 2A**). Although this model might not perfectly reflect the mechanisms regulating virus reactivation, it allowed us to investigate the function of IL-3 during viral pneumonia observed in the immunosuppressive phase of sepsis. In addition, we chose to induce a light CLP (5% of the ligated cecum) to expose the animals to only moderate inflammation and reduce mortality associated with the acute phase of sepsis as previously described (35). Seven days after the onset of sepsis, mice exhibited increased plasma IL-10 levels compared to controls, unaltered plasma TNFα levels, unaltered percentage of circulating non-inflammatory Ly6C^low^ monocytes, and reduced percentage of circulating neutrophils compared to day 4 (**Figures S4A–E**). Interestingly, we also observed an increased percentage of circulating Ly6C^high^ monocytes (**Figure S4F**), monocytes often termed “inflammatory monocytes” although their continuous recruitment was required for the resolution of inflammation in many diseases (36–38). Thus, the concomitant reduction of circulating neutrophils in combination with increased plasma IL-10 levels and increased circulating Ly6C^high^ monocytes suggest that mice exhibit an anti-inflammatory state 7 days after the onset of sepsis. Moreover, WT and *Il-3^-/-^* mice exhibited a similar phenotype after 7 days of CLP. WT and *Il-3^-/-^* mice had the same numbers of pDCs, neutrophils, B cells and CD4^+^ T cells in the lungs, spleen, liver and bone marrow (BM) (**Figures S5A–D**) and no major differences were observed in the ability of pulmonary cells to secrete TNFα, IL-10 or IL-1β after *ex vivo* stimulation with lipopolysaccharide (LPS) or CpG (**Figures S5E–G**).

Upon HSV-1 infection, CLP *Il-3^-/-^* mice were more susceptible than CLP WT mice as shown by increased mortality, pronounced weight loss and reduced body temperature over time (**Figures 2B–D**). Consistent with these findings, CLP *Il-3^-/-^* mice exhibited increased viral load compared to CLP WT mice in the BALF and lungs 3 days after HSV-1 infection (**Figure 2E**). RNA sequencing of whole lung tissue revealed that the gene transcripts upregulated in the lungs of *Il-3^-/-^* mice were associated with immune system processes, innate immune responses, defence responses to viruses and inflammatory responses (**Figures 2F, G**). RT-qPCR analyses confirmed the higher expression of genes associated with inflammation in the lungs of infected CLP *Il-3^-/-^* mice compared to CLP WT mice (**Figure 2H**), suggesting that the inability to control the viral infection observed in CLP *Il-3^-/-^* mice may result in increased lung tissue damage. Altogether, these data show that the absence of IL-3 results in reduced protection against HSV-1 infection during sepsis.

### Interleukin-3 promotes the recruitment of pDCs into the lungs

**Figure 3:**
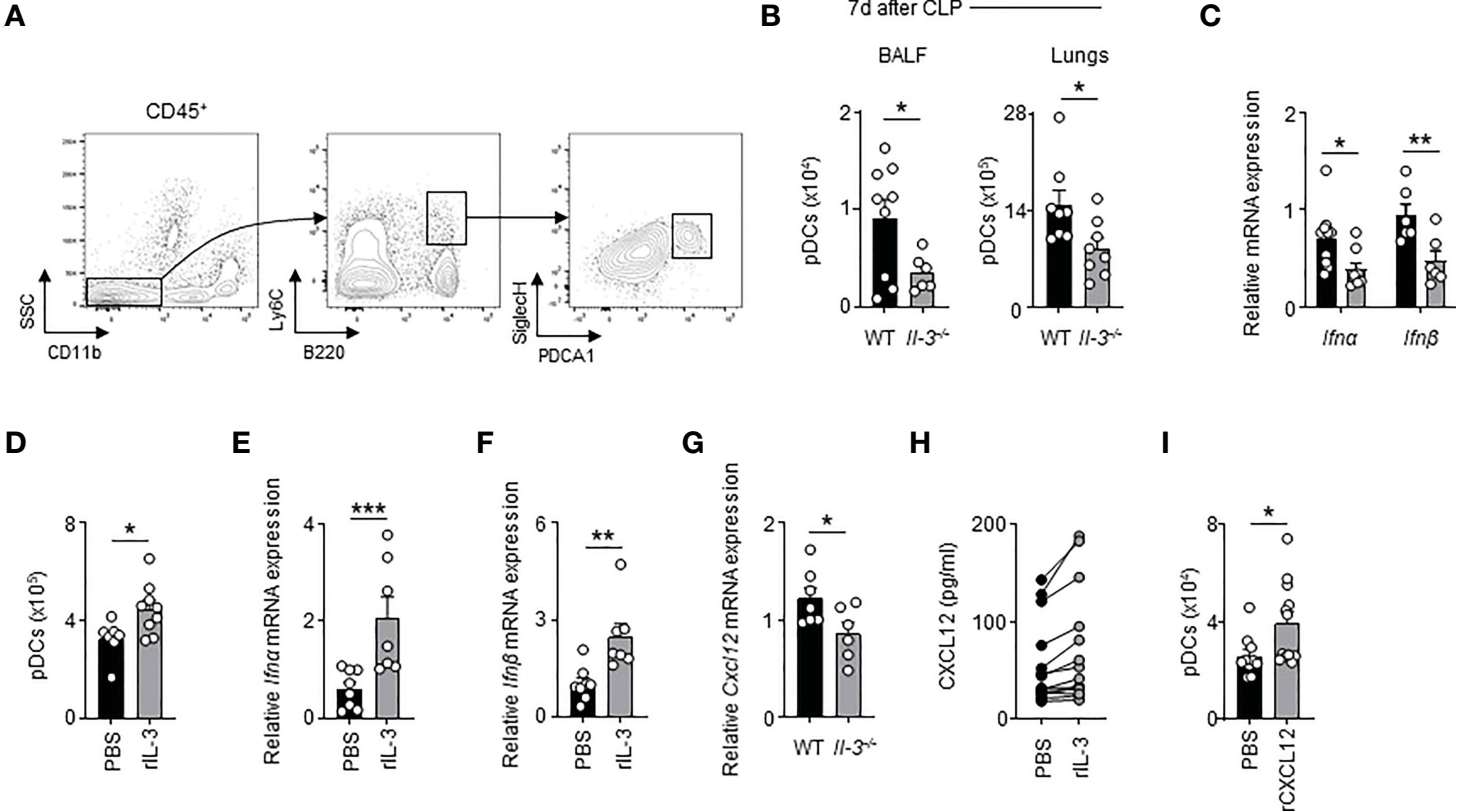
Interleukin-3 promotes the recruitment of pDC in lungs of CLP mice during viral pneumonia. **(A)** Gating strategy for murine pDCs in lungs. **(B)** Absolute number of pDCs in the BALF and lungs of CLP WT or *Il-3^-/-^* mice 3 days after HSV-1 infection (n=8-9). **(C)** Relative mRNA expression of *Ifnα* and *Ifnβ* in lungs of CLP WT or *Il-3^-/-^* mice 3 days after HSV-1 infection (n=6-10). **(D)** Absolute number of pDCs in the lungs of CLP WT mice 24h after i.n. injection of PBS or recombinant IL-3 (n=7-9). **(E, F)** Relative mRNA expression of *Ifnα* **(E)** and *Ifnβ* **(F)** in the lungs of CLP WT mice 24h after i.n. injection of PBS or recombinant IL-3 (n=7-8). **(G)** Relative mRNA expression of *Cxcl12* in the lungs of CLP WT or *Il-3^-/-^* mice 3 days after HSV-1 infection (n=6-7). **(H)** Levels of CXCL12 in the supernatant of lung cells from CLP mice 24 h after *ex vivo* stimulation with or without IL-3 (n=14). **(I)** Absolute number of pDCs in the lungs of CLP WT mice 24h after i.n. injection of PBS or recombinant CXCL12 (n=10-12). Data are mean ± s.e.m., *P < 0.05, **P < 0.01, ***P < 0.001, unpaired or paired 2-tailed Student’s t test using Welch’s correction for unequal variances was used.

Next, we investigated how IL-3 protects against viral pneumonia in sepsis. Previously, we showed that IL-3 protects against primary viral infection by promoting the recruitment of pDCs into the lungs in a CXCL12-dependent manner (21). We therefore wondered whether the same mechanism applies during viral pneumonia in sepsis. Upon HSV-1 infections, CLP *Il-3^-/-^* mice exhibited reduced numbers of PDCA-1^+^ Siglec-H^+^ CD11b^-^ B220^+^ Ly6C^+^ pDCs in BALF and lungs compared to CLP WT mice (**Figures 3A, B**). CLP *Il-3^-/-^* mice displayed also reduced mRNA expression of *Ifnα* and *Ifnβ* in lungs 3 days after HSV-1 infection (**Figure 3C**). Likewise, IL-3 administration increased the number of pDCs in the lung parenchyma of CLP WT mice as compared to controls (**Figure 3D**) and enhanced the mRNA expression of *Ifnα* and *Ifnβ* in the lungs after subsequent i.n. CpG injection (**Figures 3E, F**). Three days post-HSV-1 infection, CLP *Il-3^-/-^* mice exhibited also reduced *Cxcl12* mRNA expression in the lungs (**Figure 3G**). Moreover, CXCL12 levels were higher in the supernatant of *ex vivo* cultured lung cells derived from CLP WT mice upon IL-3 stimulation (**Figure 3H**) and intranasal injection of CXCL12 in CLP WT mice resulted in increased numbers of pDCs in the lungs (**Figure 3I**). Thus, our results suggest that IL-3 improves local antiviral defence during viral pneumonia in sepsis by increasing pDC numbers in the lungs.

### IL-3 enhances antiviral immune responses and pDC-mediated T cell immunity during sepsis

**Figure 4:**
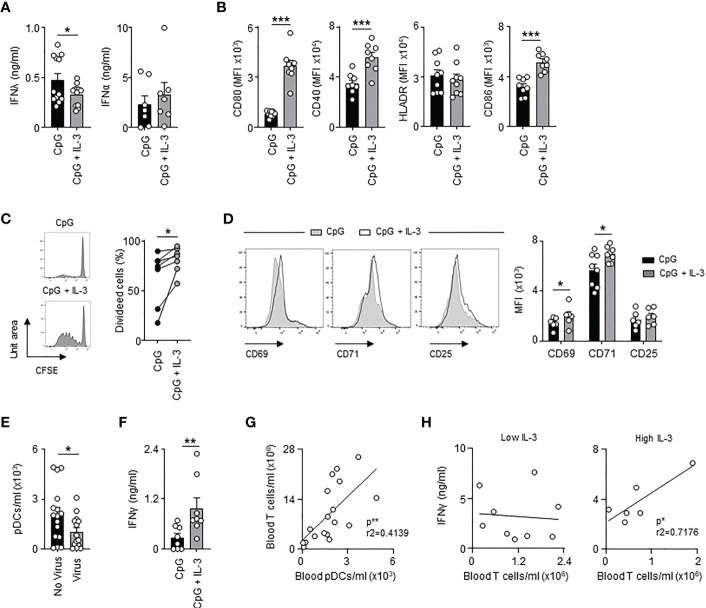
Interleukin-3 enhances pDC-mediated adaptive antiviral immunity. **(A)** Levels of IFNλ and IFNα in culture supernatant of circulating pDCs from healthy donor 24h after CpG stimulation in the presence or the absence of IL-3 (n=7-12). **(B)** Cumulative mean fluorescence intensity (MFI) of CD80, CD40, HLADR and CD86 expressed at the surface of circulating pDCs 24h after CpG stimulation in the presence or the absence of IL-3 (n=8-9). **(C–E)** Allogenic T cells were cocultured during 6 days with CpG-activated pDCs pre-treated or not with IL-3. **(C)** Representative histogram and percentage of proliferating allogenic T cells (n=7). **(D)** Representative histogram and cumulative mean fluorescence intensity (MFI) of CD69, CD71 and CD25 expressed at the surface of allogenic T cells (n=6-8). **(E)** Concentration of pDCs in blood of septic patients that developed viral pneumonia or not (n=32). **(F)** Levels of IFNγ in culture supernatant (n=8). **(G)** Correlation between the concentration of T cells and pDCs in blood of septic patients (n=18). Data were analysed by Pearson correlation test. **(H)** Correlation between plasma IFNγ levels and the concentration of T cells in blood of septic patients with low (n=9) or high (n=6) plasma IL-3 levels. Patients with low IL-3 are patients with a level of IL-3 under the mean (61.7 pg/ml) of all the patients. Patients with high IL-3 are patients with a level of IL-3 above the mean (61.7 pg/ml) of all the patients. Data were analysed by Pearson correlation test. Data are mean ± s.e.m., *P < 0.05, **P < 0.01, ***P < 0.001, paired 2-tailed Student’s t test or unpaired 2-tailed Student’s t test using Welch’s correction for unequal variances were used.

The receptor for IL-3 is expressed on human pDCs but not on murine pDCs. Therefore, we wondered whether IL-3 might also protect septic patients from viral pneumonia by directly improving the antiviral function of pDCs. pDCs are immune cells that promote antiviral immune responses either as a source of type I or type III IFNs or as antigen-presenting cells (5, 39). Interestingly, we observed that IL-3 reduced the quantity of IFNλ secreted by circulating pDCs from healthy donors 24h after *ex vivo* co-stimulation with CpG, a synthetic ligand mimicking HSV-1 infection, but had no effect on IFNα secretion (**Figure 4A**), which indicates that the increased plasma IFNλ levels observed in septic patients with high plasma IL-3 levels (**Figure S6A**) was not associated to pDCs. Flow cytometry analysis revealed that the concomitant stimulation of circulating pDCs (**Figure S6B**) by CpG and IL-3 induced increased expression of the co-stimulatory molecules CD80, CD40 and CD86 but not HLADR when compared to controls (**Figure 4B** and **Figure S6C**) suggesting that IL-3 increased the ability of pDCs to prime T cells during viral infection. Indeed, allogeneic T cells primed with CpG- and IL-3-pre-treated pDCs exhibited increased proliferation rates (**Figure 4C**) as well as increased expression levels of the activation markers CD69 and CD71 (**Figure 4D**) when compared to controls. Interestingly, only allogeneic CD4^+^ T cells showed increased expression of CD69 and CD71 after CpG- or CpG/IL-3-pre-treated pDCs priming (**Figure S7**). Septic patients with viral pneumonia (SEPICER cohort) had reduced numbers of circulating pDCs as compared to septic patients without any viral infection (**Figure 4E**). In addition, higher IFNγ levels were observed in culture supernatant of allogenic T cells primed with CpG- and IL-3-pre-treated pDCs (**Figure 4F**), IFNγ being an important component of the antiviral response (40). In septic patients, we observed that the number of circulating T cells was correlated with the number of circulating pDCs (**Figure 4G**). Likewise, the number of T cells in blood was only correlated with plasma IFNγ levels in septic patients with high plasma IL-3 levels (**Figure 4H**). Collectively, these results suggested that IL-3 enhances antiviral immune responses during sepsis by improving pDC-mediated T cell immunity.

### IL-3 improves pDC-mediated T cell immunity during SARS-CoV-2 infections

**Figure 5:**
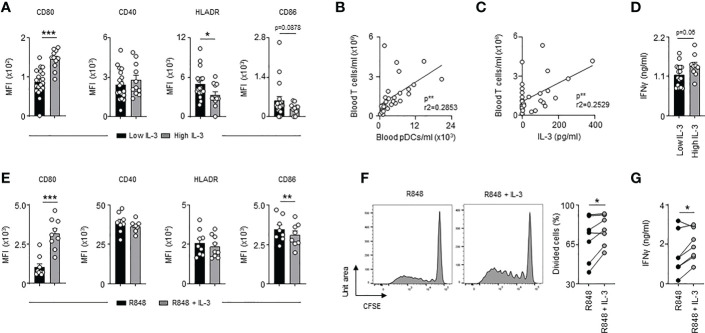
High plasma IL-3 levels in SARS-CoV-2 infections are associated with increased T cell activation. **(A)** Cumulative mean fluorescence intensity of CD80, CD40, HLADR and CD86 expressed at the surface of circulating pDCs from SARS-CoV-2^+^ patients with low or high plasma IL-3 levels (n=30). **(B)** Correlation between the concentration of blood T cells and blood pDCs in SARS-CoV-2^+^ patients (n=30). Data were analysed by Pearson correlation test. **(C)** Correlation between blood T cells concentration and plasma IL-3 levels in SARS-CoV-2^+^ patients (n=30). Data were analysed by Pearson correlation test. **(D)** Levels of plasma IFNγ in SARS-CoV-2^+^ patients with low or high plasma IL-3 levels (n=30). **(E)** Cumulative mean fluorescence intensity of CD80, CD40, HLADR and CD86 expressed at the surface of circulating pDCs 24h after R848 stimulation in the presence or the absence of IL-3 (n=8-9). **(F, G)** Allogenic T cells were cocultured during 6 days with R848-activated pDCs pre-treated or not with IL-3. **(F)** Representative histogram and percentage of proliferating allogenic T cells (n=7). **(G)** Levels of IFNγ in culture supernatant 24h after R848 stimulation in the presence or the absence of IL-3 (n=7). Data are mean ± s.e.m., *P < 0.05, **P < 0.01, ***P < 0.001, paired 2-tailed Student’s t test or unpaired 2-tailed Student’s t test using Welch’s correction for unequal variances were used.

We previously identified IL-3 as a predictive marker for severity and outcome during SARS-CoV-2 infections (21). We next investigated whether IL-3 improves adaptive antiviral immunity during coronavirus disease 2019 (COVID-19). Interestingly, SARS-CoV-2^+^ patients (**Table S6**) with high plasma IL-3 levels exhibited higher expression of CD80 and reduced expression of HLADR when compared to circulating pDCs from SARS-CoV-2^+^ patients with low plasma IL-3 levels (**Figure 5A**). The number of blood pDCs as well as the plasma IL-3 levels were both correlated with the number of blood T cells (**Figures 5B, C**). Likewise, SARS-CoV-2^+^ patients with high plasma IL-3 levels exhibited higher plasma IFNγ levels (**Figure 5D**) suggesting that IL-3-stimulated pDCs induce T cell activation during SARS-CoV-2 infections. To confirm this, we stimulated *ex vivo* circulating pDCs from healthy donors with R848, a synthetic ligand mimicking coronavirus infections, in presence or absence of IL-3. As observed in SARS-CoV-2^+^ patients (**Figure 5A**), R848-activated pDCs had higher surface expression of CD80 and reduced surface expression of CD86 in presence of IL-3 (**Figure 5E**). However, no difference was observed in the CD40 and HLADR expression suggesting that the reduced expression of HLADR on pDCs observed in SARS-CoV-2^+^ patients with high plasma IL-3 levels (**Figure 5A**) may rather reflect a different inflammatory micro-environmental state between SARS-CoV-2^+^ patients with low and high plasma IL-3 levels than a direct effect of IL-3 on the HLADR expression in circulating pDCs. Moreover, the additional stimulation of R848-activated pDCs by IL-3 resulted in an increased allogeneic T cell proliferation and higher IFNγ levels in culture supernatants 6 days after priming (**Figures 5F, G**). Collectively, our results suggested that IL-3 might also protect against SARS-CoV-2 infections by improving pDC-mediated T cell activation.

## Discussion

This study highlights a dual role of IL-3 in sepsis. IL-3 is detrimental during the acute phase by fuelling the innate immune response (19) but protects during the following immunosuppressive phase by improving antiviral defence mechanism. Such a dual role has already been described for other cytokines. Whereas type I interferons are critical for early immune responses against acute lymphocytic choriomeningitis virus (LCMV) infections, they contribute to the altered immunity during chronic LCMV infections (41, 42). Furthermore, opposite effects of IL-3 were reported during cerebral inflammation. Indeed, IL-3 limits Alzheimer’s disease by programming microglia towards an acute immune response program allowing them to clear the accumulation of β-amyloid and neurofibrillary tau in the brain (22) whereas IL-3 promotes the development of experimental autoimmune encephalitis by increasing the recruitment of leukocytes into the brain (43). Therefore, this study deepens the knowledge that IL-3 is a key mediator in inflammation and suggests that recombinant IL-3 or CD123 receptor agonists may have the potential as novel therapeutic agents during inflammatory or infectious diseases.

Innate response activator B cells and T cells have been described as the major cellular source of IL-3 in sepsis and SARS-CoV-2 infections, respectively (19, 21). The reduced plasma IL-3 levels observed in septic patients with viral pneumonia or in patients with severe SARS-CoV-2 infections might therefore reflect the lymphopenic state observed in these patients (44). Septic patients with low basal levels of plasma IL-3 at admission in the ICU would then be more susceptible to virus reactivation suggesting that plasma IL-3 levels at admission may serve as an early prognostic marker to identify patients at risk. The measurement of cytokine levels for diagnostic purposes has already been described (45, 46). Since there are new technical possibilities to determine the plasma IL-3 value in a fast, affordable and at point-of-care manner (47), clinicians could assess the risk for virus reactivation and the associated worse outcome using the SOFA score in combination with the plasma IL-3 levels and thus, adapt the therapeutic strategy.

We observed that IL-3 amplified plasmacytoid dendritic cell-mediated T cell activation upon viral activation but had no effect on type I or type III IFN production, which is consistent with a recent study showing that pDCs stimulated by IL-3 develop into a subpopulation specialized in adaptive immune function but not in type I IFN production (48). Although IL-3-stimulated pDCs were described to preferentially drive allogenic T cell differentiation into Th2 cells, our data show that IL-3-treated pDCs were associated with higher levels of IFNγ upon viral stimulation, as observed after HSV infection (30), suggesting then that IL-3 functions during viral infection as an amplifier of TLR signalling as observed in systemic lupus erythematosus (32). In mice, CLP *Il-3^-/-^* mice were more susceptible than CLP WT mice to viral pneumonia although murine pDCs do not express the α chain of the IL-3 receptor indicating that the antiviral function of IL-3 seems to work through different ways. IL-3 regulates the innate and adaptive function of pDCs during viral pneumonia by either promoting their recruitment into the airways (21) or by improving their capacity to prime T cells. IL-3 may also induce IFNλ expression, an important first-line defence against viruses in the epithelium, as IL-3 and IFNλ expressions are correlated in sepsis and SARS-CoV-2 infections (21). Considering that airway epithelial cells are one of the main orchestrators of pulmonary immune responses (49, 50), and have been identified to express the IL-3 receptor (21), IL-3 may also protect against viral pneumonia by directly stimulating pulmonary epithelial cells.

Collectively, our results revealed a new function of IL-3 during viral infection and confirmed that IL-3 may be a potential therapeutic target to control primary and secondary viral pneumonia.

## Methods

### Animals

Balb/c (Janvier, Le Genest-Saint-Isle, France) and *Il-3^-/-^* mice (Balb/c background, obtained from RIKEN BRC Laboratories, Japan) were used in this study. Majority of the mice were 8-12 weeks old when sacrificed. All animal protocols were approved by the animal review committee from the university hospital Dresden and Erlangen and the local governmental animal committee.

### Cecal ligation and puncture

The peritoneal cavity was opened during isoflurane anesthesia, and the cecum was exteriorized. To induce light-grade CLP ~10-15% of the cecum was ligated using a nonabsorbable 7-0 suture (Johnson and Johnson, New Brunswick, NJ, USA). The distal end of the cecum was then perforated using a 23 G needle, and a small drop of feces was extruded through the puncture. The cecum was relocated into the peritoneal cavity and the peritoneum was closed using a nonabsorbable 5-0 suture (Johnson and Johnson). Animals were resuscitated by s.c. injection of 1 mL of saline and pain medication (Buprenorphin, 0.15 mg/kg) was injected sc. (23G Terumo, Leuven, Belgium). Mice were infected with HSV-1 after 7 days of sepsis.

### Temperature

The temperature of each animal was measured by rectal insertion of a temperature sensor while the mouse was under anesthesia.

### Mouse infection

CLP mice were anesthetized with isoflurane and infected intra-nasally with 15x10^6^ PFU HSV-1 in a volume of 15µl saline, 8 μg of CpG (Enzo Life Sciences, Farmingdale, NY, USA), 400 ng of recombinant IL-3 (R&D Systems, Minneapolis, MN, USA) or 500 ng of recombinant CXCL12 (Peprotech, Rocky Hill, NJ, USA).

### Murine leukocytes isolation

Peripheral blood was collected by heart puncture using heparin (Ratiopharm, Ulm, Germany) as anticoagulant. Erythrocytes were lysed using Lysis Buffer (BD Biosciences, San Jose, CA, USA). After organ harvest, single cell suspensions were obtained as follows: perfused lungs and liver were cut in small pieces and subjected to enzymatic digestion with 450 U/ml collagenase I (Sigma Aldrich), 125 U/ml collagenese IX (Sigma Adrich), 60 U/ml hyaluronidase (Sigma Aldrich), 60 U/ml Dnase (Sigma Aldrich) and 20 mM Hepes (Thermo Fisher Scientific, Waltham, MA, USA) for 1 hour at 37°C while shaking. Spleens were homogenized through a 40 μm nylon mesh and bone marrow (BM) cells were flushed out of the femurs and tibias. Broncho-alveolar lavage (BAL) was performed by flushing the lungs with 2 × 1 ml of PBS to retrieve the infiltrated and resident leukocytes. Total viable cell numbers were obtained using Trypan Blue (Carl Roth).

### Lung cells stimulation *in vitro*

Lung cell suspensions from CLP mice were cultured in RPMI-1640 GlutaMax supplemented with 10% FCS, 25 mM of Hepes, 1 mM sodium pyruvate, 100U/ml of Penicillin–Streptomycin, and 20 μg/ml of Gentamicin at 37°C in the presence of 5% CO2. Lung cell suspensions were stimulated in 12-well plates (10^6^ cells/ml) during 24 h by IL-3 (20 ng/ml).

### Human leukocyte isolation

*From healthy donor:* Peripheral blood samples were obtained from leukoreduction system (LRS)-chamber of healthy donors through the Department of Transfusion medicine (Erlangen, Germany). Then, peripheral blood mononuclear cells were isolated by Ficoll density gradient (GE Healthcare, Little Chalfont, UK). pDCs were purified by negative selection using Diamond pDC Isolation Kit II (Miltenyi Biotec, Bergisch Gladbach, Germany) and T cells were purified by positive selection using anti-CD3 microbeads (Miltenyi Biotec), according to the manufacturer’s instructions. *From septic and SARS-CoV-2^+^ patients:* After blood collection, plasma of all study participants was immediately obtained by centrifugation, transferred into cryotubes, and stored at −80°C until further processing. Red blood cells were then lysed using Lysing buffer (BD Biosciences), according to the manufacturer’s instructions. For flow cytometry analysis, leukocytes from SARS-CoV-2^+^ patients were fixed 1 h after the staining with BD Cytofix buffer (BD Biosciences) in order to inactivate the virus.

### pDC and T cell activation

Purified pDCs were cultured in RPMI-1640 GlutaMax supplemented with 10% fetal calf serum (FCS) and 100U/ml of Penicillin-Streptomycin at 37°C in the presence of 5% CO2. pDCs were stimulated (in 96-well plates at 5x10^4^ cells/ml) by CpG (4 µg/mL) (Miltenyi Biotec), R848 (10 µg/mL) (*In vivo*gen, Toulouse, France) or recombinant IL-3 (40 ng/mL) (R&D systems). After 24h, pDCs were stained for phenotypic analysis, or collected for the mixed lymphocyte reaction (MLR), or their supernatants were collected for cytokine measurement. For MLR experiment, activated pDCs were washed once with PBS and then co-cultured for 6 days in 96-well plates with allogenic T cells stained with 5μM of CFSE (Biolegend) (pDC: T cell ratio 1:5). Then, T cells were stained for phenotypic analysis or their supernatants were collected for cytokine measurement.

### Quantitative RT-PCR

Real-time PCR was performed as previously described (51). Briefly, RNA was extracted from whole tissue by RNeasy mini kit (Qiagen, Venlo, Netherlands). Complementary DNA was reverse transcribed from 1 µg total RNA with Moloney murine leukemia virus reverse transcriptase (Thermo Fisher Scientific) using random hexamer oligonucleotides for priming (Thermo Fisher Scientific). The amplification was performed with a Biorad CFX-Connect Real-time-System (Thermo Fisher Scientific) using the SYBR Green (Eurogentec, Seraing, Belgium) or TaqMan (Thermo Fisher Scientific) detection system. Data were analyzed using the software supplied with the Sequence Detector (Life Technologies). The mRNA content was normalized to the hypoxanthine-guanine phosphoribosyltransferase (*Hprt*) mRNA for mouse genes. Gene expression was quantified using the ΔΔCt method.

### Cytokine detection

Mouse: Secreted TNFα (Biolegend, San Diego, CA, USA), IL-10 (Biolegend), IL-1β (Biolegend), IFNλ (R&D systems), IFNβ (Biolegend) and IFNα (R&D systems) were measured by ELISA according to the manufacturer’s instructions. Human: Secreted IFNλ (R&D Systems), IFNγ (Biolegend), IL-10 (Biolegend) and TNFα (Biolegend) were measured by enzyme linked immunosorbent assay (ELISA) according to the manufacturer’s instructions. Quantification of human IL-3 (R&D Systems) was performed in combination with chemiluminescent detection (R&D Systems) for increased sensitivity. The assays were performed according to the manufacturer’s instructions and measured in a microplate reader set to luminescence mode (BMG Labtech, Ortenberg, Germany) with an integration time of 2 seconds per well, yielding a sensitivity of 3.9 pg/ml IL-3.

### RNA sequencing

RNA was sequenced on an Illumina HiSeq 2500 as external service (Eurofins Genomics Germany GmbH, Ebersberg, Germany). *Reads processing and mapping:*Initial quality control using FastQC (52) was performed for the available RNA-seq datasets. Subsequent processing included filtering with SortMeRNA (53) to remove contaminants of ribosomal RNA as well as trimming of short or low-quality reads by Trimmomatic (39) software. For main processing the remaining reads were mapped to Mus musculus release M17 (GRCm38.p6) reference genome available from the GENCODE project (https://www.gencodegenes.org) using STAR (54) alignment software. Comprehensive gene annotation on the primary assembly (chromosomes and scaffolds) was chosen as superset of the main annotation. Unambiguously mapped and unique reads were kept. SAMtools (55) was used to convert the resulting sequence alignment maps to sorted binary alignment format (BAM) for downstream analysis. *Differential expression, gene-ontology term analyses and barplots:* Feature counting was performed using HTSeq (56) for all replicates against the respective release M17 gene transfer file. DESeq2 (57) was used in R environment (58) for differential expression analysis of count data. The differentially expressed genes identified were filtered to results with absolute linear fold change values above a threshold of 1.5 and Benjamini-Hochberg procedure FDR adjusted p-values below 0.05. Over-represented GO-terms were identified by use of DAVID (Database for Annotation, Visualization and Integrated Discovery) web service (59, 60) collectively as well as separately for both up- and down-regulated gene sets. GO-term barplots (**Figure 2G**) display the top 10 over-represented GO-term results in respect of attributed p-values for the full set of dis-regulated genes and the sub-selection of up-regulated genes respectively. *GO-term-dependent gene-selection:* Library-size normalized count data of filtered differentially expressed genes were restricted to those which were contained in identified GO-terms. Genes contained within the GO term “Inflammatory response” were selectively annotated in **Figure 2F** [Volcano plot imm. infl. resp.] in case of differential regulation. The sequencing raw data are accessible under the following link: https://www.ncbi.nlm.nih.gov/geo/query/acc.cgi?acc=GSE224299.

### Flow cytometry

The following antibodies were used for flow cytometric analyses: Mouse: anti-CD317-BV650 (927; Biolegend), anti-Ly6C-FITC (AL-21; BD Biosciences), anti-B220-BUV737 (RA3-6B3; BD Biosciences), anti-CD11c-PerCP Cy5.5 (HL3; Biolegend), anti-CD11b-PE CF594 (M1/70; BD Biosciences), anti-F4/80-BV510 (T45-2342; BD Biosciences), anti-Ly6G-BUV395 (1A8; BD Biosciences), anti-SiglecH-Pacific Blue (551; BD Biosciences), anti-CD45.2-BV786 (104; BD Biosciences), anti-MHCII-BV711 (M5/114.15.2; BD Biosciences). Human: anti-CD16-FITC (3G8; Biolegend), anti-CD64-PE (10.1; Biolegend), anti-CD303-PerCP Cy5.5 (201A; Biolegend), anti-CD123-PE CF594 (7G3; BD Biosciences), anti-CD45-BV786 (HI30; Biosciences), anti-CD11c-BV711 (B-ly6; BD Biosciences), anti-CD20-BV650 (2H7; BD Biosciences), anti-CD15-PE (W6D3; BD Biosciences), anti-CD3-BV510 (UCHT1; BD Biosciences), anti-HLADR-BUV395 (G46-6; BD Biosciences), anti-CD14-BUV737 (M5E2; BD Biosciences), anti-CD80-PE (L307.4; BD Biosciences), anti-CD86-BV510 (FUN-1; BD Biosciences), anti-CD40-BUV737 (5C3; BD Biosciences), anti-CD3-BUV395 (SK7; BD Biosciences), anti-CD69-BV421 (FN50; BD Biosciences), anti-CD71-BV650 (M-A712; BD Biosciences), anti-CD25-BV711 (M-A251; BD Biosciences), anti CD4-PerCP Cy5.5 (RPA-T4; BD Biosciences) and anti-CD8-PE CF594 (RPA-T8; BD Biosciences). Data were acquired on a Celesta (BD Biosciences) flow cytometer and analyzed with FlowJo 10 (FlowJo LLC, Ashland, OR, USA).

### Virus preparation and titration

Preparation of virus stocks was performed using a modified protocol described by Sodeik et al. (Sodeik et al., 1997). Briefly, subconfluent BHK-21 cells were infected with RPMI 1640 supplemented with 20 mM Hepes (5 ml/175 cm2 flask) containing a low multiplicity of infection (MOI; 0.01). After 1–2 h, 20 ml D10 medium was added and cells were cultivated for 3–4 d, until complete cytopathic effect was observed. Medium was harvested, cell debris was removed *via* centrifugation at 2,575 g and 4°C for 10 min, and virus containing supernatant was centrifuged at 39,742 g at 4°C for 2 h. Virus pellets were overlaid with a small volume of PBS at 4°C overnight. Afterwards, virus pellets were resuspended, aliquoted, and stored at −80°C until further use.

### Plaque assay

Vero cells, used for viral titration, were cultured in D10 medium (DMEM; Lonza) supplemented with 10% FCS (Merck), 2 mM L-glutamine, 100 U/ml penicillin, and 100 mg/ml streptomycin. Titration of BALF and lung cell suspensions in different dilutions was performed using Vero cells at 100% of confluency. Cells were washed with RPMI 1640 (Lonza, Basel, Switzerland) supplemented with 0.1% BSA (Sigma-Aldrich) and 20 mM Hepes (Lonza) before 200 µl BALF and lung cell suspensions was added. After incubation on a rocking platform for 1 h at room temperature, the inoculum was removed, and 400 µl D10 medium containing 10 µg/ml human IgG (Sigma-Aldrich) were added to each well. Cells were cultured in an incubator for 3 d until visible plaques had formed. Media were discarded, and the cells were fixed with 250 µl of 9% formaldehyde in PBS for 10 min. Afterwards, the formaldehyde solution was removed and 200 µl crystal violet solution (5% crystal violet in ethanol, 1:50 dilution in H2O) was added and incubated for 10 min. Subsequently, wells were washed with water and air-dried. Finally, plaques were counted, and the viral titer was calculated and indicated in PFUs per milliliter.

### Human specimen

*Human data from secondary analyses of patients participating in the VISS-trial* (German Clinical Trials Register: DRKS00000505). The VISS-trial was first approved by the local ethics committee (Trial-Code-Nr.: S058-2009) on June, 8th 2009, and was conducted in the surgical intensive care unit of the University Hospital of Heidelberg, Germany. In total, 60 patients within the VISS-cohort, classified according to the criteria of the International Sepsis Definitions Conference, were enrolled with an onset of sepsis syndrome ≤ 24 hours. 1 patient from the published VISS-trial was excluded from the retrospective analysis because it was a significant outlier for plasma IL-3 levels. Written informed consents were obtained from the study patients or their legal designees. Patients were eligible for enrollment with an onset of sepsis syndrome within 24 h. The initial blood draw was also performed within this period. In contrast, patients with an onset of sepsis syndrome > 24 h were excluded from the study. The management of patients with septic shock in the intensive care unit included early goal-directed therapy (according to Rivers and colleagues), elimination of the septic focus, and broad-spectrum antibiotics. Patients with preexisting immunosuppressive diseases were excluded from the study. Blood samples (EDTA) as well as deep tracheal secretion samples from patients with septic shock were collected after the diagnosis of sepsis at sepsis onset (day 0), as well as 1, 7, 14, 21, and 28 days (VISS-trial) later. Afterwards, the virologic diagnostics were performed as previously described (61) and correlated to the clinical outcome. For the presented IL-3 measurements an amendment was submitted to the local ethics committee which was approved on November, 22th 2013. *Human data from prospective measurements and analyses of patients participating in the SEPICER-trial.* The SEPICER-trial was first approved by the local ethics committee on February 1, 2021 (UKER 459_20B), and was conducted in the surgical intensive care unit of the University Hospital of Erlangen, Germany. In total, 32 septic patients and 30 patients positive for SARS-CoV-2 PCR from oral swabs, oral fluid, or BALF were enrolled in this trial. Patients with low IL-3 are patients with a level of IL-3 under the mean (61.7 pg/ml) of all the patients. Patients with high IL-3 are patients with a level of IL-3 above the mean (61.7 pg/ml) of all the patients.

### Statistics

Statistics and significant outliers were determined using GraphPad Prism 7.0 software. Results were expressed as mean ± S.E.M. and expressed as identified in legends. For comparing 2 groups, statistical tests included paired 2-tailed Student’s t test, Wilcoxon test or unpaired 2-tailed Student’s t test using Welch’s correction for unequal variances were used. Univariate and multivariate logistic regression analysis were performed using IBM SPSS Statistics 28.0.1 software. P values of 0.05 or less were considered to denote significance.

## Data availability statement

The data presented in the study are deposited in the GEO repository and accessible via https://www.ncbi.nlm.nih.gov/geo/query/acc.cgi?acc=GSE224299.

## Ethics statement

The studies involving human participants were reviewed and approved by the local ethics committee. The VISS-trial (Trial-Code-Nr.: S058-2009 on June, 8th 2009) was conducted in the surgical intensive care unit of the University Hospital of Heidelberg, Germany. The SEPICER-trial (UKER 459_20B on February 1, 2021) was conducted in the surgical intensive care unit of the University Hospital of Erlangen, Germany. The patients/participants provided their written informed consent to participate in this study. The animal studies were reviewed and approved by the local animal review committee from the university hospital Dresden and Erlangen and the local governmental animal committee.

## Author contributions

AB designed and performed experiments, analysed and interpreted the data, made the figures, and wrote the manuscript. FH designed and performed experiments, analysed and interpreted the data, made the figures, and performed statistical analysis. FU, BK, IS, DS, PM-Z performed experiments. FC, AM, AJ, PD, MP, AA, AS, MW, MAW, TB, CK and RG provided clinical samples and intellectual input; GW conceived and supervised the project, designed experiments, interpreted data, made the figures, and wrote the manuscript. All authors edited the manuscript. All authors contributed to the article and approved the submitted version.

## Supplementary material

Supplementary Figure 1 Markers of immunosuppression in septic patients. **(A, B)** Analysis of plasma IL-10 **(A)** and TNFα **(B)** levels from healthy donors and septic patients (SEPIL-3 trial, n=40) on days 7, 14, and 28 after sepsis onset (n=20). **(C)** HLA-DR expression on circulating CD14^+^ monocytes from healthy donors and on days 7, 14, and 28 after sepsis onset (n=20). **(D–F)** Sub-lethal sepsis was induced in WT mice using the CLP model. The abdomen of control mice (sham) was opened, the caecum was moved out the abdominal and replaced inside, and the abdomen was closed. Control mice were killed 2 days after surgery. **(D, E)** Plasma levels of IL-10 **(D)** and TNFα **(E)** (n=5-6). **(F)** Percentage of Ly6C^-/low^ monocytes in blood (n=6). Data are mean ± s.e.m., *P < 0.05, unpaired, 2-tailed Student’s t test using Welch’s correction for unequal variances was used.

Supplementary Figure 2 Interleukin-3 is associated with reduced viral infections in septic patients in the prospective SEPICER trial. Levels of IL-3 in the plasma of septic patients positive for SARS-CoV-2 (n=9), HSV (n=3) in the tracheal secretion or in septic patient without viral pneumonia (n=17). Data are mean ± s.e.m., *P < 0.05, **P < 0.01, unpaired, 2-tailed Student’s t test using Welch’s correction for unequal variances was used.

Supplementary Figure 3 Risk for virus reactivation during sepsis according to IL-3 and SOFA score. Plasma IL-3 levels (left) and SOFA score (right) of septic patients (pooled VISS and SEPICER cohorts) with low, intermediate, and high risk to develop virus reactivation in lungs (n=72). Data are mean ± s.e.m., *P < 0.05, **P < 0.01, ***P < 0.001, Tukey’s multiple comparison test was used.

Supplementary Figure 4 Markers of immunosuppression in CLP mice. **(A–D)** Sub-lethal sepsis was induced in WT mice using the CLP model. The abdomen of control mice (sham) was opened, the caecum was moved out the abdominal and replaced inside, and the abdomen was closed. Control mice were killed 2 days after surgery. **(A, B)** Plasma levels of IL-10 **(A)** and TNFα **(B)** (n=5-6). **(C)** Gating strategy for murine circulating neutrophils, Ly6C^high^ monocytes, and Ly6C^low^ monocytes. **(D)** Percentage of Ly6C^low^ monocytes in blood (n=6). **(E)** Percentage of neutrophils in blood (n=6). **(F)** Percentage of Ly6C^high^ monocytes in blood (n=6). Data are mean ± s.e.m., *P < 0.05, unpaired, 2-tailed Student’s t test using Welch’s correction for unequal variances was used.

Supplementary Figure 5 WT mice exhibit the same phenotype than *Il-3^-/-^* mice 7 days after a light CLP. Sub-lethal sepsis was induced in WT and *Il-3^-/-^* mice using the CLP model. Mice were sacrificed 7 days later. **(A–D)** Absolute number of pDCs **(A)**, neutrophils **(B)**, B cells **(C)** and CD4^+^ T cells **(D)** in the lungs, spleen, liver and BM of CLP WT and *Il-3^-/-^* mice (n=6-11). **(E–G)** Levels of TNFα **(E)**, IL-10 **(F)** and IL-1β **(G)** in the supernatant of pulmonary cells from CLP WT and *Il-3^-/-^* mice stimulated *ex vivo* for 3 days with PBS, LPS or CpG (n=6). Data are mean ± s.e.m., *P < 0.05, unpaired, 2-tailed Student’s t test using Welch’s correction for unequal variances was used.

Supplementary Figure 6 Phenotype of CpG-activated pDCs upon IL-3 *ex vivo* stimulation. **(A)** Levels of plasma IFNλ in septic patients with high or low plasma IL-3 levels (n=32). **(B)** Gating strategy of pDCs in humans. **(C)** Representative histogram of MFI for CD80, CD40, HLADR and CD86 expressed at the surface of circulating pDCs 24h after CpG stimulation in the presence or the absence of IL-3. Data are mean ± s.e.m., *P < 0.05, unpaired, 2-tailed Student’s t test using Welch’s correction for unequal variances was used.

Supplementary Figure 7 Interleukin-3 enhances pDC-mediated CD4^+^ T cell activation. Representative histogram and cumulative mean fluorescence intensity (MFI) of CD69, CD71 and CD25 expressed at the surface of allogenic CD4^+^ and CD8^+^ T cells primed with CpG- or CpG/IL-3-pre-treated pDCs (n=6-8). Data are mean ± s.e.m., *P < 0.05, paired 2-tailed Student’s t test was used.
