# Supplementary figures and images for: Interleukin-3 protects against viral pneumonia in sepsis by enhancing plasmacytoid dendritic cell recruitment into the lungs and T cell priming
Source: Front Immunol. 2023 Feb 22;14:1140630. doi: 10.3389/fimmu.2023.1140630 (PMC9996195; doi:10.3389/fimmu.2023.1140630)

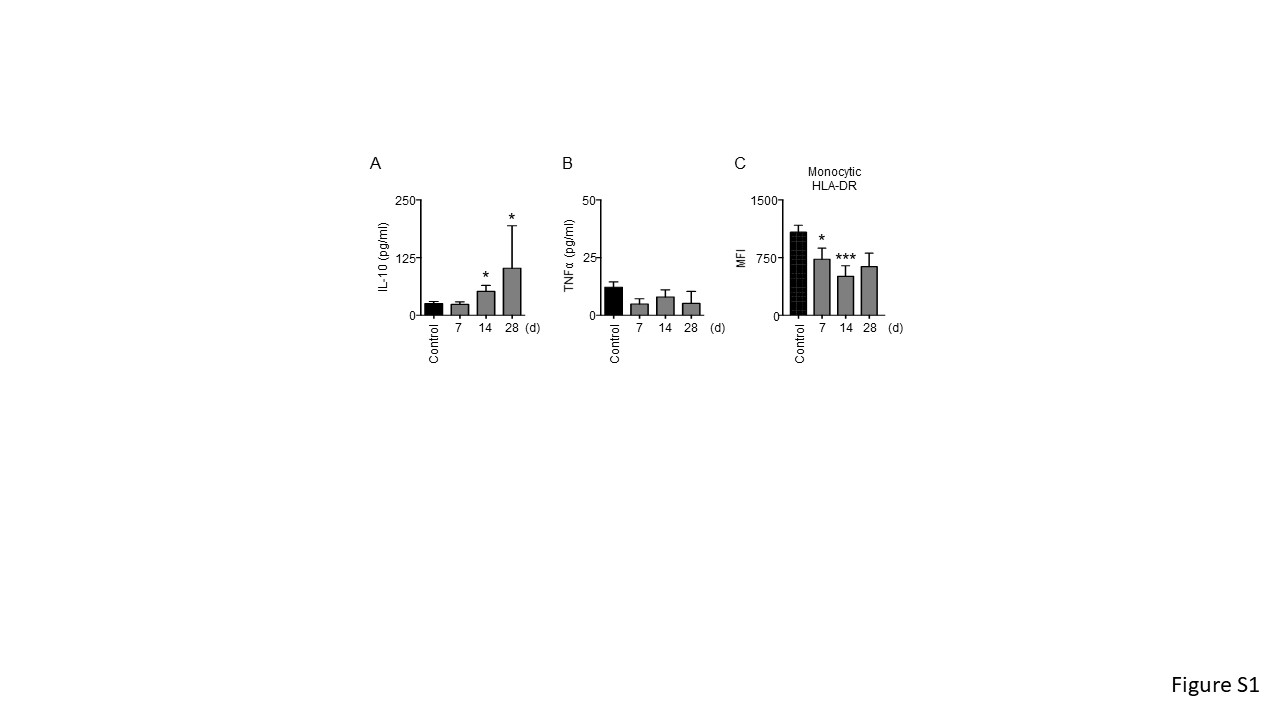

Supplement: Supplementary Figure 1 — Markers of immunosuppression in septic patients. (A, B) Analysis of plasma IL-10 (A) and TNFα (B) levels from healthy donors and septic patients (SEPIL-3 trial, n=40) on days 7, 14, and 28 after sepsis onset (n=20). (C) HLA-DR expression on circulating CD14+ monocytes from healthy donors and on days 7, 14, and 28 after sepsis onset (n=20). (D–F) Sub-lethal sepsis was induced in WT mice using the CLP model. The abdomen of control mice (sham) was opened, the caecum was moved out the abdominal and replaced inside, and the abdomen was closed. Control mice were killed 2 days after surgery. (D, E) Plasma levels of IL-10 (D) and TNFα (E) (n=5-6). (F) Percentage of Ly6C-/low monocytes in blood (n=6). Data are mean ± s.e.m., *P < 0.05, unpaired, 2-tailed Student’s t test using Welch’s correction for unequal variances was used. [file Image_1.jpeg]

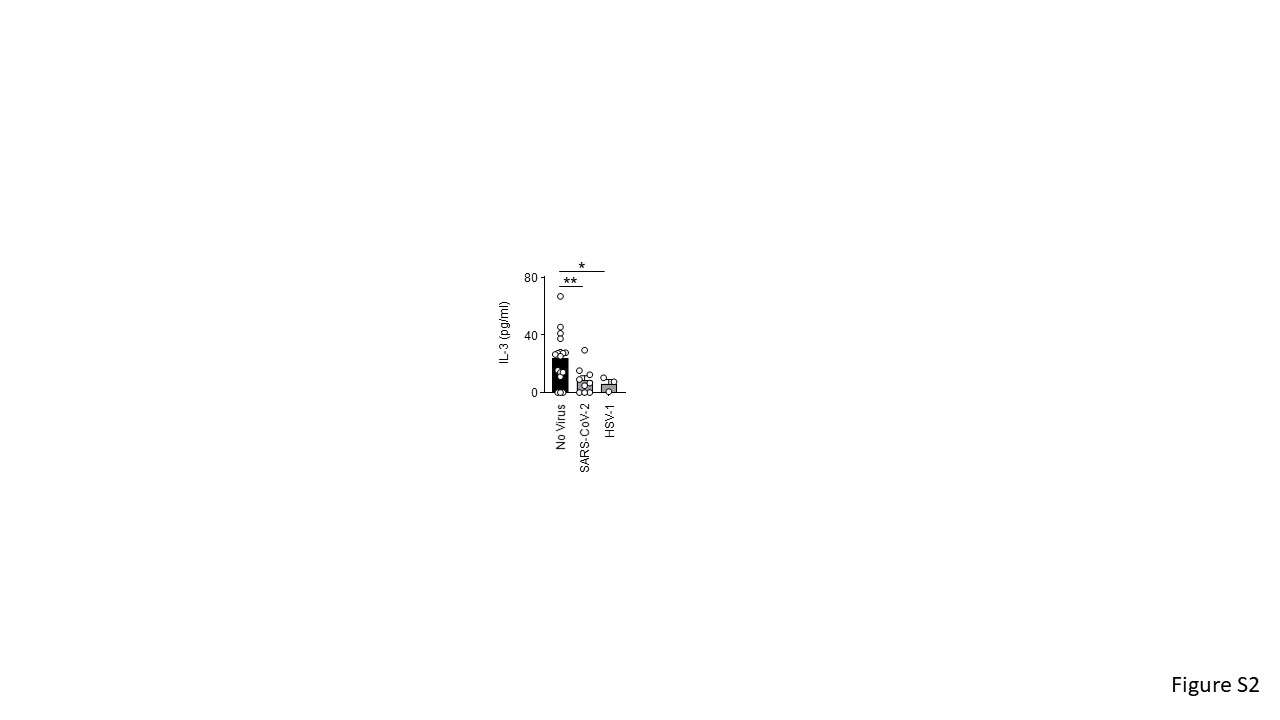

Supplement: Supplementary Figure 2 — Interleukin-3 is associated with reduced viral infections in septic patients in the prospective SEPICER trial. Levels of IL-3 in the plasma of septic patients positive for SARS-CoV-2 (n=9), HSV (n=3) in the tracheal secretion or in septic patient without viral pneumonia (n=17). Data are mean ± s.e.m., *P < 0.05, **P < 0.01, unpaired, 2-tailed Student’s t test using Welch’s correction for unequal variances was used. [file Image_2.jpeg]

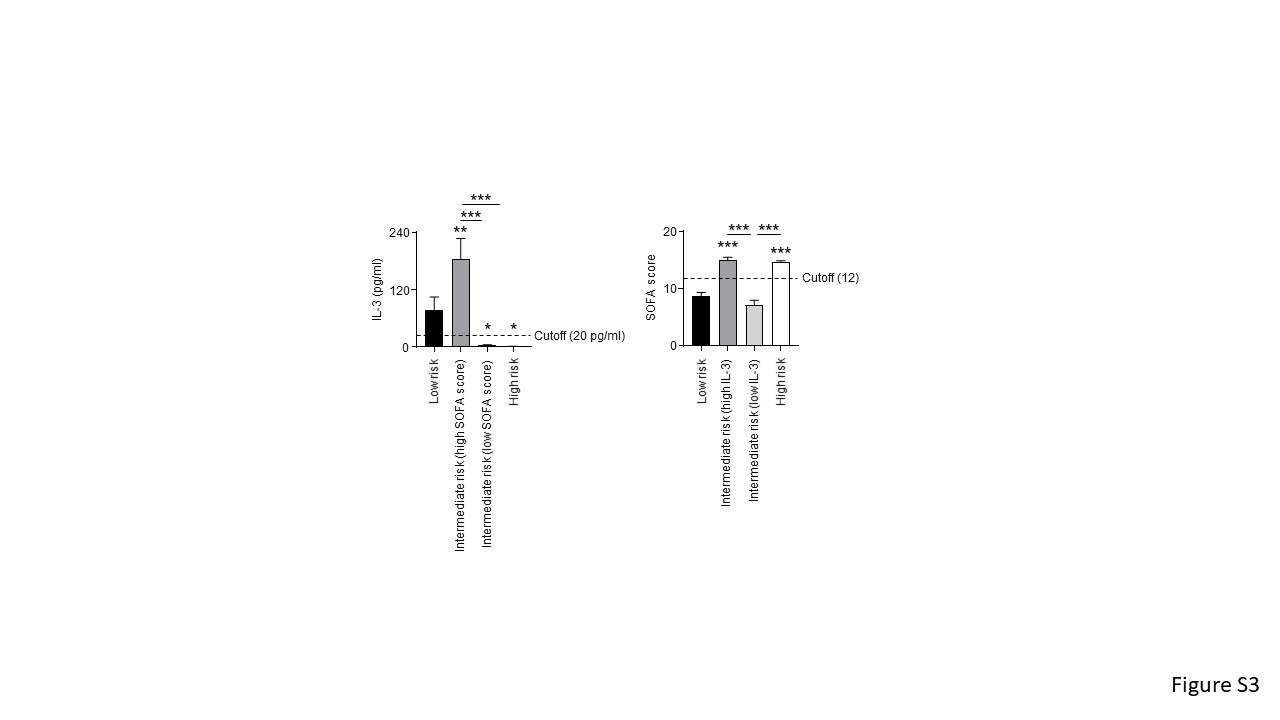

Supplement: Supplementary Figure 3 — Risk for virus reactivation during sepsis according to IL-3 and SOFA score. Plasma IL-3 levels (left) and SOFA score (right) of septic patients (pooled VISS and SEPICER cohorts) with low, intermediate, and high risk to develop virus reactivation in lungs (n=72). Data are mean ± s.e.m., *P < 0.05, **P < 0.01, ***P < 0.001, Tukey’s multiple comparison test was used. [file Image_3.jpeg]

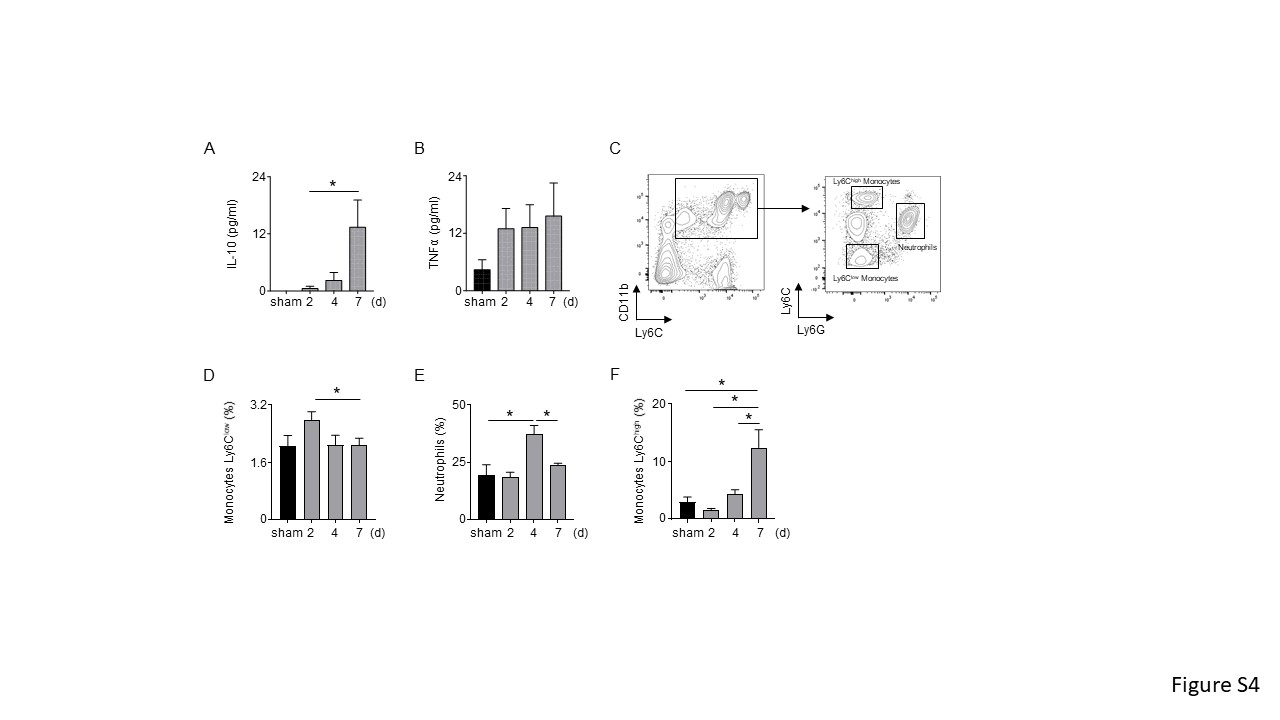

Supplement: Supplementary Figure 4 — Markers of immunosuppression in CLP mice. (A–D) Sub-lethal sepsis was induced in WT mice using the CLP model. The abdomen of control mice (sham) was opened, the caecum was moved out the abdominal and replaced inside, and the abdomen was closed. Control mice were killed 2 days after surgery. (A, B) Plasma levels of IL-10 (A) and TNFα (B) (n=5-6). (C) Gating strategy for murine circulating neutrophils, Ly6Chigh monocytes, and Ly6Clow monocytes. (D) Percentage of Ly6Clow monocytes in blood (n=6). (E) Percentage of neutrophils in blood (n=6). (F) Percentage of Ly6Chigh monocytes in blood (n=6). Data are mean ± s.e.m., *P < 0.05, unpaired, 2-tailed Student’s t test using Welch’s correction for unequal variances was used. [file Image_4.jpeg]

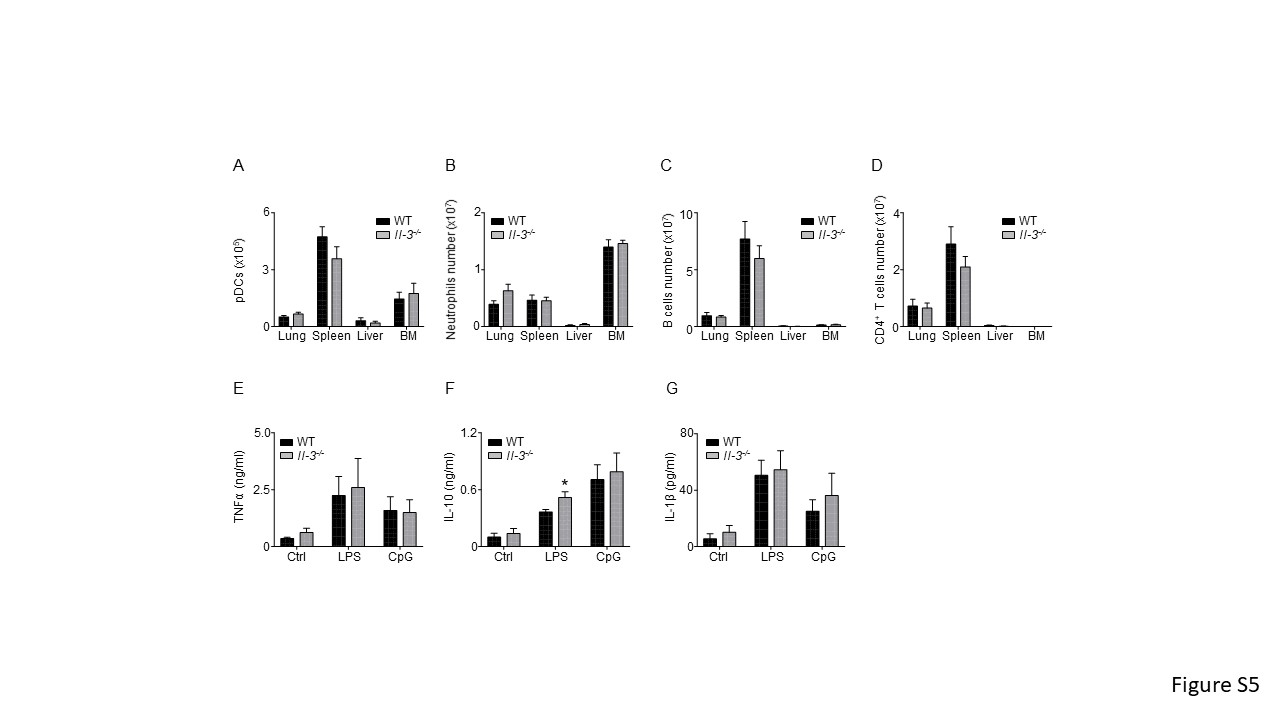

Supplement: Supplementary Figure 5 — WT mice exhibit the same phenotype than Il-3-/- mice 7 days after a light CLP. Sub-lethal sepsis was induced in WT and Il-3-/- mice using the CLP model. Mice were sacrificed 7 days later. (A–D) Absolute number of pDCs (A), neutrophils (B), B cells (C) and CD4+ T cells (D) in the lungs, spleen, liver and BM of CLP WT and Il-3-/- mice (n=6-11). (E–G) Levels of TNFα (E), IL-10 (F) and IL-1β (G) in the supernatant of pulmonary cells from CLP WT and Il-3-/- mice stimulated ex vivo for 3 days with PBS, LPS or CpG (n=6). Data are mean ± s.e.m., *P < 0.05, unpaired, 2-tailed Student’s t test using Welch’s correction for unequal variances was used. [file Image_5.jpeg]

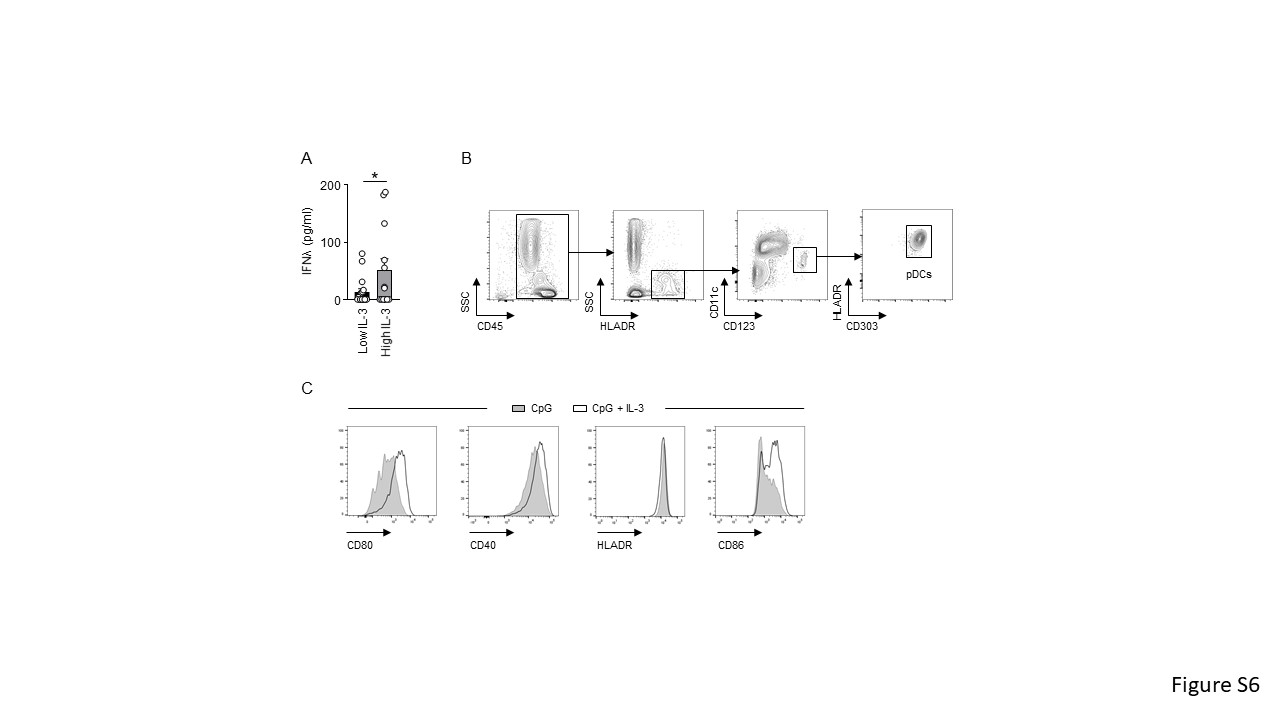

Supplement: Supplementary Figure 6 — Phenotype of CpG-activated pDCs upon IL-3 ex vivo stimulation. (A) Levels of plasma IFNλ in septic patients with high or low plasma IL-3 levels (n=32). (B) Gating strategy of pDCs in humans. (C) Representative histogram of MFI for CD80, CD40, HLADR and CD86 expressed at the surface of circulating pDCs 24h after CpG stimulation in the presence or the absence of IL-3. Data are mean ± s.e.m., *P < 0.05, unpaired, 2-tailed Student’s t test using Welch’s correction for unequal variances was used. [file Image_6.jpeg]

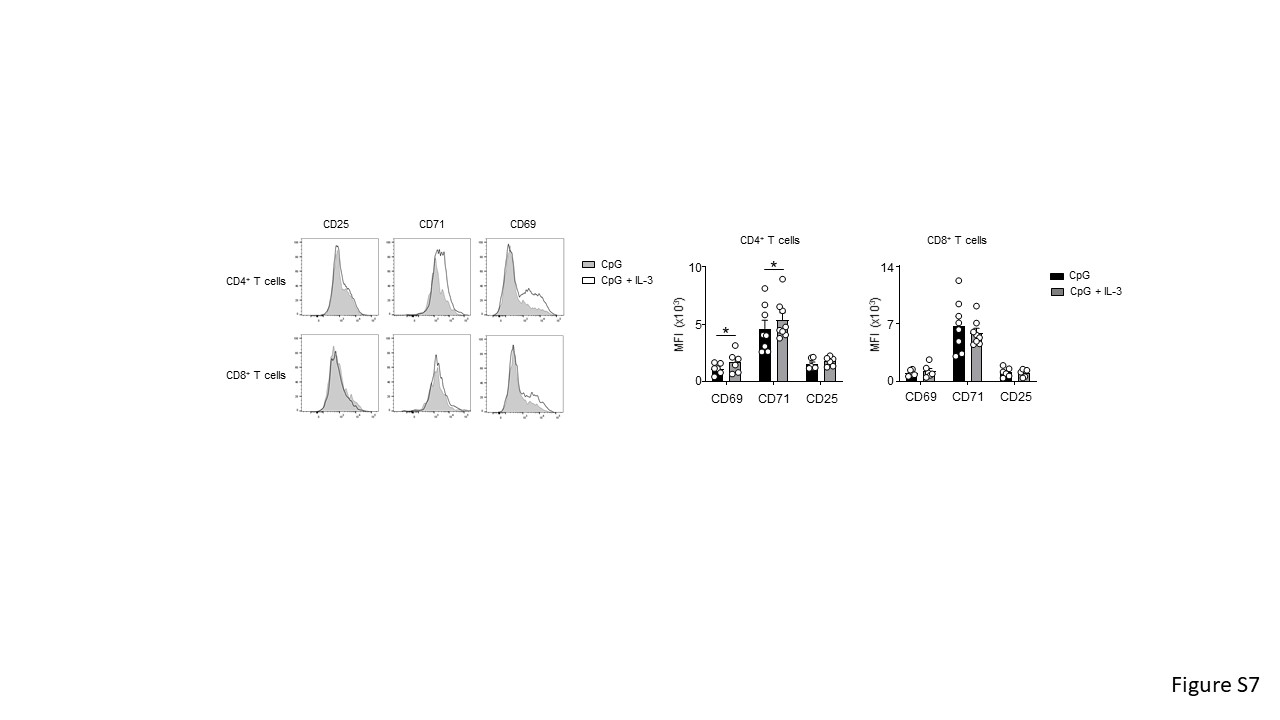

Supplement: Supplementary Figure 7 — Interleukin-3 enhances pDC-mediated CD4+ T cell activation. Representative histogram and cumulative mean fluorescence intensity (MFI) of CD69, CD71 and CD25 expressed at the surface of allogenic CD4+ and CD8+ T cells primed with CpG- or CpG/IL-3-pre-treated pDCs (n=6-8). Data are mean ± s.e.m., *P < 0.05, paired 2-tailed Student’s t test was used. [file Image_7.jpeg]
