# Supplementary material for: Interleukin-3 protects against viral pneumonia in sepsis by enhancing plasmacytoid dendritic cell recruitment into the lungs and T cell priming
Source: Front Immunol. 2023 Feb 22;14:1140630. doi: 10.3389/fimmu.2023.1140630 (PMC9996195; doi:10.3389/fimmu.2023.1140630)
Supplement: Supplementary file 8 [file Table_1.docx]

**Table S1: Percentage of patients tested positive for HSV or CMV 0, 7, 14, 21 and 28 days after the sepsis onset in the HSV^+^CMV^-^, HSV^-^CMV^+^ and HSV^+^CMV^+^ cohorts.**

|  | **day 0** | **day 7** | **day 14** | **day 21** | **day 28** |
| --- | --- | --- | --- | --- | --- |
| **HSV^+^ CMV^-^** | 25% | 50% | 15% | 10% | 0% |
| **HSV^-^ CMV^+^** | 75% | 0% | 0% | 0% | 25% |
| **HSV^+^ CMV^+^** | 33.3% (HSV)  33.3% (CMV) | 44.4% (HSV)  11.1% (CMV) | 11.1% (HSV)  33.3% (CMV) | 11.1% (HSV)  22.2% (CMV) | 0% (HSV)  0% (CMV) |
