# Supplementary material for: Interleukin-3 protects against viral pneumonia in sepsis by enhancing plasmacytoid dendritic cell recruitment into the lungs and T cell priming
Source: Front Immunol. 2023 Feb 22;14:1140630. doi: 10.3389/fimmu.2023.1140630 (PMC9996195; doi:10.3389/fimmu.2023.1140630)
Supplement: Supplementary file 9 [file Table_2.docx]

**Table S2: Baseline data of septic patients in our prospective SEPICER cohort (n=32).**

| **Baseline data of septic patients (n=32)** | |
| --- | --- |
| **Demographic data**  Age, y  Male sex | 66.5 (± 10.0)  24 (75%) |
| **Clinical characteristics**  Death  Septic shock  Viral pneumonia  SOFA-Score  Plasma Interleukin-3 level (pg/ml)  Time of blood collection (days)   - Non-infected cohort - SARS-CoV-2 cohort - HSV cohort | 14 (44%)  21 (66%)  14 (44%)  7 (± 3)  20 (± 18)  8.3 (± 9.9)  15.9 (± 13.2)  7 (± 2.8) |
| **Main diagnosis**  Primary respiratory  Trauma  Intra-abdominal inflammation  Ischaemic colitis  Colon perforation  Pancreatitis  Peritonitis  Liver abscess  Acute cholecystitis  Primary abdominal gas gangrene  Cancer  Esophageal Cancer  Ovarial Carcinoma  Cervix Cancer  Cholangiocarcinoma  Fournier´s gangrene  Unknown | 10 (31%)  3 (9%)  3 (9%)  2 (6%)  2 (6%)  2 (6%)  1 (3%)  1 (3%)  1 (3%)  1 (3%)  1 (3%)  1 (3%)  1 (3%)  1 (3%)  2 (6%) |

Data is presented as the number (%) or the mean (± standard deviation).

Baseline data of the septic patients in the VISS-cohort are already published (refer to reference 30).
