# Supplementary material for: Interleukin-3 protects against viral pneumonia in sepsis by enhancing plasmacytoid dendritic cell recruitment into the lungs and T cell priming
Source: Front Immunol. 2023 Feb 22;14:1140630. doi: 10.3389/fimmu.2023.1140630 (PMC9996195; doi:10.3389/fimmu.2023.1140630)
Supplement: Supplementary file 10 [file Table_3.docx]

**Table S3: Cutoff threshold of the SOFA score based on the development of lung viral infection in septic patients.** Both cohorts were pooled. Patients with primary viral infection and with a collection day later than 7 days after sepsis onset were excluded from the analysis. The 2-tailed minimal *p*-value approach (chi-square test; n=72) as used for analysis. The optimal cutoff value with the lowest p-value and the highest probability of viral reactivation is marked in grey.

| SOFA score | *p*-value (chi-sqare test) | SOFA | N | Probability of virus reactivation |
| --- | --- | --- | --- | --- |
| ≥16 | 0.53 | Low  High | 60  12 | 48.3%  58.3% |
| ≥14 | 0.06 | Low  High | 42  30 | 40.5%  63.3% |
| ≥12 | **< 0.01** | **Low**  **High** | **30**  **42** | **26.7%**  **66.7%** |
| ≥10 | **< 0.01** | **Low**  **High** | **21**  **51** | **23.8%**  **60.8%** |
| ≥8 | **0.02** | **Low**  **High** | **11**  **61** | **18.2%**  **55.7%** |
| ≥6 | **0.02** | **Low**  **High** | **5**  **67** | **0.0%**  **53.7%** |

Bold values are significant (*p* < 0.05).
