# Supplementary material for: Interleukin-3 protects against viral pneumonia in sepsis by enhancing plasmacytoid dendritic cell recruitment into the lungs and T cell priming
Source: Front Immunol. 2023 Feb 22;14:1140630. doi: 10.3389/fimmu.2023.1140630 (PMC9996195; doi:10.3389/fimmu.2023.1140630)
Supplement: Supplementary file 11 [file Table_4.docx]

**Table S4:** **Multivariate analysis of the impact of different risk factors for virus reactivation during sepsis (n=72).**

|  | **Univariate** | **Multivariate** | |
| --- | --- | --- | --- |
|  | ***p*-value** | **CI** | **p-value** |
| **Age ≥ 65 years** | 0.564 |  |  |
| **Survival** | 0.805 |  |  |
| **Sex** | **0.016** | 0.014 – 0.497 | **0.038** |
| **SOFA-score ≥ 12 *** | **< 0.001** | 0.157 – 0.588 | **< 0.001** |
| **IL-3 < 20 pg/ml **** | **0.046** | 0.007 – 0.456 | **0.043** |

*cutoff was determined using *p*-value approach.

**cutoff was selected based on the previous published study about IL-3 in Sars-CoV-2 infections (refer to reference 21); bold values are significant (*p* < 0.05).
