# Supplementary material for: Interleukin-3 protects against viral pneumonia in sepsis by enhancing plasmacytoid dendritic cell recruitment into the lungs and T cell priming
Source: Front Immunol. 2023 Feb 22;14:1140630. doi: 10.3389/fimmu.2023.1140630 (PMC9996195; doi:10.3389/fimmu.2023.1140630)
Supplement: Supplementary file 12 [file Table_5.docx]

**Table S5: Risk for virus reactivation during sepsis according to risk groups:** Low (n=10; IL-3 ≥ 20 pg/ml and SOFA < 12) and intermediate (n=34; IL-3 ≥ 20 pg/ml and SOFA ≥12 or IL-3 < 20 pg/ml and SOFA < 12) vs. high (n=28; IL-3 < 20 pg/ml and SOFA ≥ 12).

|  | Viral infec. | Viral infec. | OR | 95%-CI |
| --- | --- | --- | --- | --- |
| Low risk | 2/10 (20.0%) | 14/44 (31.8%) | - | - |
| Intermediate risk | 12/34 (35.3%) |  |  |  |
| High risk | 22/28 (78.6%) | 22/28 (78.6%) | 7.857 | 2.607 – 23.682 |
