# Supplementary material for: Interleukin-3 protects against viral pneumonia in sepsis by enhancing plasmacytoid dendritic cell recruitment into the lungs and T cell priming
Source: Front Immunol. 2023 Feb 22;14:1140630. doi: 10.3389/fimmu.2023.1140630 (PMC9996195; doi:10.3389/fimmu.2023.1140630)
Supplement: Supplementary file 13 [file Table_6.docx]

**Table S6: Baseline data of SARS-CoV-2^+^ patients (n=30).**

| **Baseline data of SARS-CoV-2^+^ patients (n=30)** | |
| --- | --- |
| **Demographic data**  Age, y  Male sex | 62.0 (± 9.8)  22 (73%) |
| **Clinical characteristics**  Death  Stay in hospital (d)  Ventilation  Time of ventilation (d)  CRP value (mg/dl) | 9 (30%)  58 (± 48)  24 (80%)  37 (± 25)  121 (± 102) |
| Viral load (CT value) | 31 (± 6) |
| Plasma Interleukin-3 level (pg/ml) | 62 (± 92) |

Data is presented as the number (%) or the mean (± standard deviation).
